# Nanostructural Arrangements and Surface Morphology on Ureasil-Polyether Films Loaded with Dexamethasone Acetate

**DOI:** 10.3390/nano11061362

**Published:** 2021-05-21

**Authors:** João Augusto Oshiro-Junior, Angelo Lusuardi, Elena M. Beamud, Leila Aparecida Chiavacci, M. Teresa Cuberes

**Affiliations:** 1 Department of Applied Mechanics and Project Engineering, Mining and Industrial Engineering School of Almaden, University of Castilla-La Mancha, Plaza Manuel Meca 1, 13400 Almadén, Spain; joaooshiro@yahoo.com.br (J.A.O.-J.); angelo.lusuardi@gmail.com (A.L.); elenamaria.beamud@uclm.es (E.M.B.); 2 Laboratory of Development and Characterization of Pharmaceutical Products, Department of Pharmacy, Center for Biological and Health Sciences, State University of Paraíba (UEPB), Campina Grande, Paraíba 58429-600, Brazil; 3 Department of Drugs and Medicines, School of Pharmaceutical Sciences, São Paulo State University (UNESP), Highway Araraquara-Jaú, Araraquara 14800-903, Brazil; leila.chiavacci@unesp.br

**Keywords:** organic-inorganic hybrid films, atomic force microscopy, ultrasonic force microscopy, sol-gel

## Abstract

Ureasil-Poly(ethylene oxide) (u-PEO500) and ureasil-Poly(propylene oxide) (u-PPO400) films, unloaded and loaded with dexamethasone acetate (DMA), have been investigated by carrying out atomic force microscopy (AFM), ultrasonic force microscopy (UFM), contact-angle, and drug release experiments. In addition, X-ray diffraction, small angle X-ray scattering, and infrared spectroscopy have provided essential information to understand the films’ structural organization. Our results reveal that while in u-PEO500 DMA occupies sites near the ether oxygen and remains absent from the film surface, in u-PPO400 new crystalline phases are formed when DMA is loaded, which show up as ~30–100 nm in diameter rounded clusters aligned along a well-defined direction, presumably related to the one defined by the characteristic polymer ropes distinguished on the surface of the unloaded u-PPO film; occasionally, larger needle-shaped DMA crystals are also observed. UFM reveals that in the unloaded u-PPO matrix the polymer ropes are made up of strands, which in turn consist of aligned ~180 nm in diameter stiffer rounded clusters possibly formed by siloxane-node aggregates; the new crystalline phases may grow in-between the strands when the drug is loaded. The results illustrate the potential of AFM-based procedures, in combination with additional physico-chemical techniques, to picture the nanostructural arrangements in polymer matrices intended for drug delivery.

## 1. Introduction

Ureasil-polyether hybrid films provide an extremely versatile matrix—platform for many different applications, including controlled drug delivery [1,2,3]. These materials consist of polyether macromers, such as Poly(ethylene oxide) (u-PEO) or Poly(propylene oxide) (u-PPO), linked by urea bridges to a silicate backbone. They can be prepared using the sol-gel method, with superb processability, mechanical, thermal, and chemical stability, luminescence, and biocompatibility. Their properties can be tailored using polyether macromers of different molecular weight [4], or blends of them [5].

In this study, we have prepared u-PEO and u-PPO films from polyether macromers of relatively small molecular weight (500 and 400, respectively), loaded them with dexamethasone acetate (DMA), and performed drug release experiments, with the aim of obtaining information about the conformation of their networks and how it is modified when the drug is incorporated and released. To this purpose, a series of physico-chemical techniques [6] such as X-ray diffraction (XRD), small-angle X-ray scattering (SAXS), and Fourier-transformed infrared spectroscopy (FT-IR) have been applied to the films’ characterization. Contact-angle studies have also been performed, and particular attention has been devoted to the investigation of the films using atomic force microscopy (AFM)-based procedures, including ultrasonic force microscopy (UFM), which is a relatively new technique, extremely powerful for mapping surface and subsurface stiffness inhomogeneities [7,8,9].

The incorporation and release of several drugs from ureasil-polyether matrices has already been reported in the literature. Sodium diclofenac was incorporated into u-PEO hybrid matrices [10]. The release profile of antitumor cisplatin molecules and cisplatin-derived species from ureasil-polyether matrices have been investigated [11,12]. Triamcinolone release has also been analyzed [13]. Recently, release studies from human intragenic antimicrobial peptides from ureasil-polyether matrices were carried out [14].

To the best of our knowledge, the incorporation and release of dexamethasone acetate from ureasil-polyether matrices has not yet been studied, although it has been studied from other matrices [15,16,17,18]. Dexamethasone is an anti-inflammatory steroid drug, the utility of which in treating COVID-19 disease, in addition to vaccines, is currently under investigation [19].

The research conducted has provided us with a wealth of information on the interaction of DMA within ureasil-polyether matrices. The interrelation of data from different techniques allows for a better understanding of the nanostructural organization within organic-inorganic hybrid matrices, providing invaluable insight into aspects that may influence sustained-release technology.

## 2. Materials and Methods

### 2.1. Preparation of the Ureasil-Polyether Hybrid Materials

The ureasil-polyether hybrid materials were synthesized by the well-known sol-gel process. Briefly, a precursor was prepared from a functionalized polyether, based on Poly(ethylene oxide) (NH_2_-PEO-NH_2_) of molecular weight 500 g·mol^−1^ (for u-PEO) and based on Poly(propylene oxide) (NH_2_-PPO-NH_2_) of molecular weight 400 g·mol^−1^ (for u-PPO) dissolved in ethanol [1,2]. A modified alkoxide, 3-(isocyanatopropyl)-triethoxysilane (IsoTrEOS) (Sigma-Aldrich, São Paolo, Brasil 95% purity, CAS #24801-88-5) in a polymer/alkoxide molar ratio of 1:2 was added to this solution, and the resulting solution was maintained at reflux for 24 h at 60 °C to promote the formation of the hybrid precursor (EtO)_3_Si(CH_2_)_3_NHC(=O)NHCHCH_3_CH_2_-(polyether)-CH_2_CH_3_CHNH(O=)NHC(CH_2_)_3_Si(OEt)_3._ Subsequently, the solvent was removed using a rotary evaporator (IKA RV 10, Staufen, Germany) operated at 60 °C and 175 mbar.

To prepare the films, the precursor was dissolved in water and ethanol in an appropriate vessel, and HCl was added as a catalyst to subject the precursor to the sol-gel hydrolysis and condensation reactions, in the proportion 500 µL ethanol, 25 µL water, and 25 µL HCl catalyst to 0.75 mg of ureasil-polyether hybrid precursor. To load the drug, crystalline DMA powder (dexametasona acetate micro, SM Empreendimentos Farmaceuticos Lta São Paolo, Brasil, CAS: 1177-87-3) in 3% wt/wt proportion to the precursor in our case was dissolved in the ethanol/water solution, and the precursor and then the HCl catalyst were added to induce the reactions. Films of ~1 mm thickness were typically prepared.

### 2.2. In Vitro Drug Released

The u-PEO500 and u-PPO400 films were immersed in 900 mL of medium (phosphate buffer 7.2 pH with 0.5% of procetyl AWS^®^ (CRODA, Rawcliffe Bridge, UK)) to guarantee the sink condition at 37 ± 0.5 °C and were stirred with a USP dissolution apparatus 2 (paddle) at a speed of 50 rpm. At time intervals, 5 mL of filtered release medium was removed for analysis and replaced with the same volume of medium. The DMA amount in the extracted solutions was analyzed by UV-vis absorbance at 241 nm, using a UV-Vis Cary 60 Spectrophotometer (Agilent Technologies, Melbourne, Australia). The cumulative percentage of drug release was calculated from the average of three parallel monitoring. The results were expressed as the mean ± SD of three experiments. All results obtained in the in vitro drug release study are presented as means and standard deviations (SD). The results were compared by ANOVA and post-hoc Tukey. The significance level (p) adopted was 0.05. Statistical analyses were performed with the program Instat for Windows (GraphPads software, San Diego, USA). Drug release kinetics was analyzed by plotting the mean release data versus time, which were fitted with different mathematical models [20]. In all cases the SigmaPlot 10.0 program (Systat Software Inc, San Jose, CA, USA) was used.

### 2.3. X-Ray Difraction

X-ray Diffraction measurements were performed in an equipment Philips X’Pert MPD (Eindhoven, Holland), using CuK α radiation (1.54056 Å) with 40 KV and 40 mA. It incorporates 0.04 rad soller slits for both incident and diffracted beams, an automatic 12.5 mm programmable divergence slit, and a Xe gas sealed proportional detector. Data were collected in an angular range between 1° and 50° (2θ) with a step size of 0.01° and a counting time of 0.70 s per step. The data analysis was carried out with the Fityk software (Varsaw, Poland) [21] (open source).

### 2.4. Small Angle X-ray Scattering

SAXS measurements were performed at the NCD beamline of ALBA Synchrotron (Barcelona, Spain). The beamline was equipped with a 2D Pilatus 300 k detector located 910.9 mm from the sample, recording the image of the scattering intensity, *I(q)*, as a function of the modulus of the scattering vector, $q=4\pi /(\lambda \sin\left(\frac{\varepsilon }{2}\right))$, where ε is the X-ray scattering angle. The data were normalized considering the varying intensity of the direct X-ray beam, the detector sensitivity, and the sample transmission. The GSAS-II software (Argonne, IL, USA) [22] (open source) was used.

### 2.5. Fourrier-Transformed Infrared Spectroscopy

FTIR spectra (4 cm^−1^ resolution, wavenumber range 500–4000 cm^−1^) were recorded using a Shimadzu IRPrestige-21 spectrometer (Tokio, Japan), using the ATR method. Small pieces of the ureasil-polyether hybrid films (≈1 mm thick) were cut and placed in the instrument sample holder. The data were acquired and analyzed using the software Shimadzu IR solution 1.21 (Tokio, Japan).

### 2.6. Scanning Probe Microscopy

Contact-mode atomic force microscopy (AFM), lateral force microscopy (LFM) and ultrasonic force microscopy (UFM) were performed using Brucker Multimode III (Santa Barbara, CA) (AFM/LFM) and NANOTEC (Madrid, Spain) (AFM/LFM and UFM [7]) instruments). For UFM, ultrasonic frequencies of ~3.8 MHz and modulation frequencies of 2.4 KHz were applied from a piezoelectric element placed under the sample. Typically, Olympus Silicon Nitride cantilevers with a nominal spring constant of 0.06 N/m and a nominal tip radius of 20 nm were used. The measurements were performed in air, at ambient conditions. Data analysis was performed with WSxM software (Madrid, Spain) [23].

### 2.7. Contact-Angle (Wettability)

Wettability tests (hydrophobic/hydrophilic) were performed based on the contact angle of water droplets on ureasil-polyether hybrid films. Droplets of 10 µL water were applied at a rate of 2 µL/s, using a 15+ OCA (Dataphysics) apparatus and SCA software 20.2.0 (DataPhysics Instruments GmbH, Filderstadt, Germany), evaluating the measurements after 15 s. Film samples ~2 cm in diameter and ~1 mm thick were used, and the experiments were carried out at room temperature.

## 3. Results and Discussion

**Figure 1:**
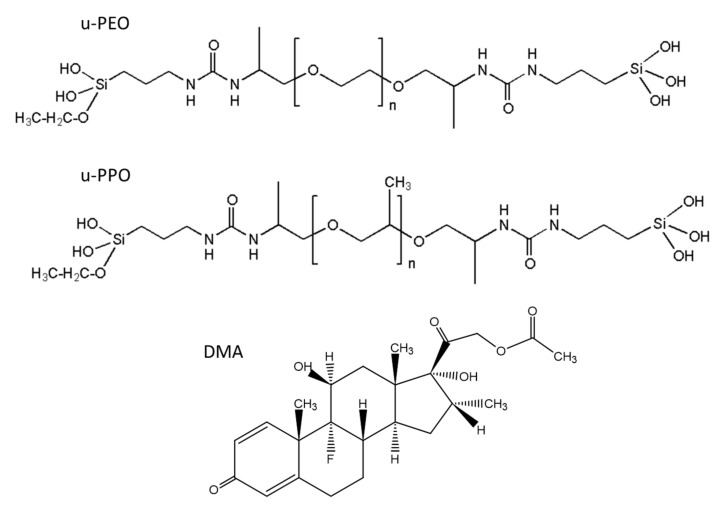
Structural formulas of u-PEO, u-PPO, and dexamethasone acetate.

Figure 1 shows the structural formulas of the precursor molecules of u-PEO and u-PPO, and DMA. For u-PEO500, the PEO chain length contains less than n = 12 oxyethylene units, and for u-PPO400, the PPO chain length contains less than n = 7 oxypolypropylene units. As hydrolysis and condensation reactions take place during the sol-gel process, the silanol terminal groups of different molecules interact with each other to form the inorganic siloxane nodes that create the matrix network. Besides, interactions among the urea and polyether moieties from different molecules may also occur. In particular, in low molecular weight ureasil-polyether like ours, the number of urea-urea linkages is expected to be quite large [4]. The formation of hydrogen-bonded urea-polyether associations is also possible, as the N-H groups of the urethane linkages are donor sites, and the ether oxygens, hydrogen bond acceptors. As the DMA molecules dissolve together with the precursor molecules when preparing the films, they may also interact and/or influence the sol-gel reactions leading to film formation [13].

**Figure 2:**
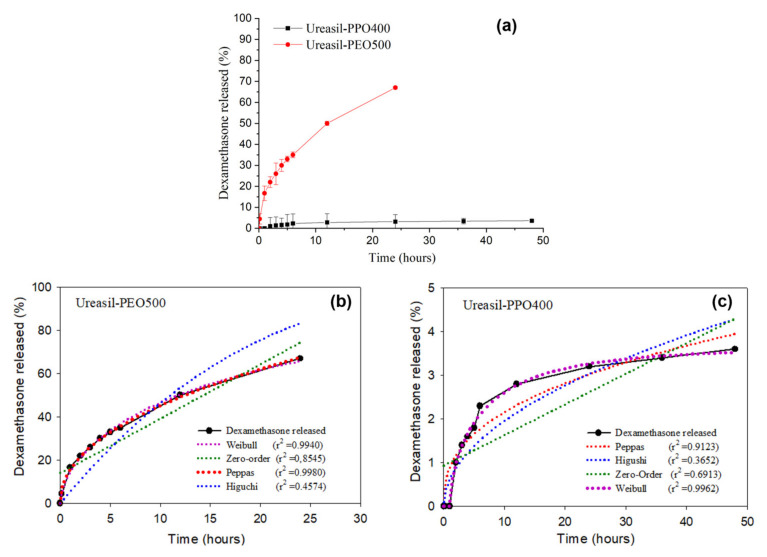
(**a**) In vitro dissolution profiles of DMA from u-PEO500 and u-PPO400 films. (**b**,**c**) Fittings of different mathematical models to the DMA release profile from (**b**) u-PEO500 and (**c**) u-PPO400.

Figure 2 presents the release profiles of DMA-loaded u-PEO500 and u-PPO400 films. Figure 2a allows us to visualize the different release rates of the films. Figure 2b,c show the different mathematical models applied to fit the DMA release profile in each case.

As it is clearly noticeable from Figure 2a, the samples prepared with u-PEO500 exhibit much faster release rates than those prepared with u-PPO400. Typically, release from u-PEO matrices is much faster than from u-PPO matrices, because in hydrophilic u-PEO the drug molecules can easily diffuse into the release medium through the free volume of the swollen network [1,2]. Whereas u-PEO has a highly hydrophilic character, the presence of the additional methyl group in u-PPO decreases its hydrophilicity. As a result, its affinity for the dissolution medium is decreased, and a lower relaxation of the polymer chains and a lower degree of swelling of the u-PPO matrix is expected. In our case, u-PEO500 and u-PPO400 hybrid materials have similar molecular weight, so the molecular weight of the polymer chain is not a factor in determining the different release profiles.

Fitting drug release data using mathematical models provides a tool to elucidate the main transport mechanisms that control the drug release process [20,24,25,26,27]. In Figure 2b,c Higuchi [28], Peppas [29] and Weibull [30] models have been considered. The criterion used to choose among these models is the statistical coefficient of determination (r^2^), which is used to evaluate the fit of the model equation. According to the values of r^2^, the DMA release from u-PEO500 (Figure 2b) fits best with the Peppas model, while that of u-PPO400 (Figure 2c) fits best with the Weibull model.

The Peppas model is based on a power law correlation between drug release and time ($\left(release\;of\;DMA\right)=constant\cdot {\left(time\right)}^{n}$). For the Peppas model the values of exponent n determine the mechanism of drug transport out of matrix [29]. When values of *n* are less than 0.45, release is expected to occur by Fickian diffusion, whereas values of n between 0.45 ˂ *n* ˂ 0.89 suggest that the release is governed by anomalous transport, involving both matrix swelling and drug diffusion. In a swelling-controlled release mechanism, release depends mostly on solvent penetration. For u-PEO500, the value of the exponent *n* is 0.4574, indicating that the release of the drug from the matrix to the medium presumably occurs by anomalous transport.

The Weibull model is an empirical approach, not based in any kinetic theory $\left(\left(release\;of\;DMA\right)=1-\exp[-constant\cdot {\left(time\right)}^{b}]\right)$. Nevertheless, reports in the literature indicate that the Weibul model does provide information about the drug release process, with the value of the exponent b correlated with the mechanism of drug transport out of the matrix [30]. When the value of *b* is less than 0.75, release is expected to occur by Fickian diffusion. The u-PPO presented a b exponent value of 0.7409. Therefore, the release of DMA from DMA-loaded u-PPO400 presumably occurs mostly by Fickian diffusion.

**Figure 3:**
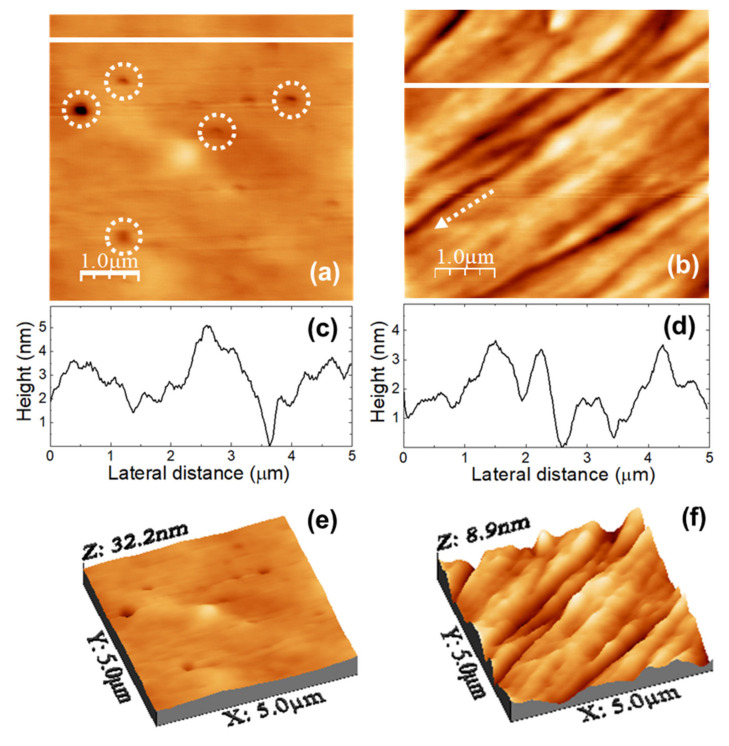
(**a**,**b**) Contact mode AFM topographic images of (**a**) unloaded u-PEO500 and (**b**) unloaded u-PPO400 films. (**c**,**d**) Height-contours profiles along the white lines in (**a**,**b**), respectively. (**e**,**f**) 3D representations of (**a**,**b**), respectively.

Figure 3 shows the topography of (5000 × 5000) nm surface areas recorded on unloaded (a) u-POE500 and (b) u-PPO400 films using contact-mode AFM. Figure 3c,d correspond to height contour profiles along the continuous white lines in Figure 3a,b, respectively. Figure 3e,f are 3D representations of Figure 3a,b. The surface of the u-PEO500 film is characterized by scattered pores as large as ≈200 nm in diameter; some of the pores have been pointed out with dashed white circles in Figure 3a, and are apparent in Figure 3e. On the u-PPO400 films’ surfaces polymer “ropes” are apparent (see Figure 3b,f) oriented along a well-defined direction, indicated by a dashed white arrow in Figure 3b. The root mean square (RMS) roughness on Figure 3a (u-PEO500) and Figure 3b (u-PPO400) is of 1.80 nm and 1.35 nm, respectively. The presence of pores causes the colour-scale range in the u-PEO image to be much larger than in the u-PPO image (see Figure 3e,f). Nevertheless, in the areas where there are no pores, the surface corrugation is only slightly larger on the u-PEO than on the u-PPO surface, as can be seen in Figure 3c,d. The pores favor the penetration of water into the matrix, leading to swelling, and thus modifying the surface morphology. The presence of pores helps to explain the higher drug release rates in u-PEO than in u-PPO films [31].

**Figure 4:**
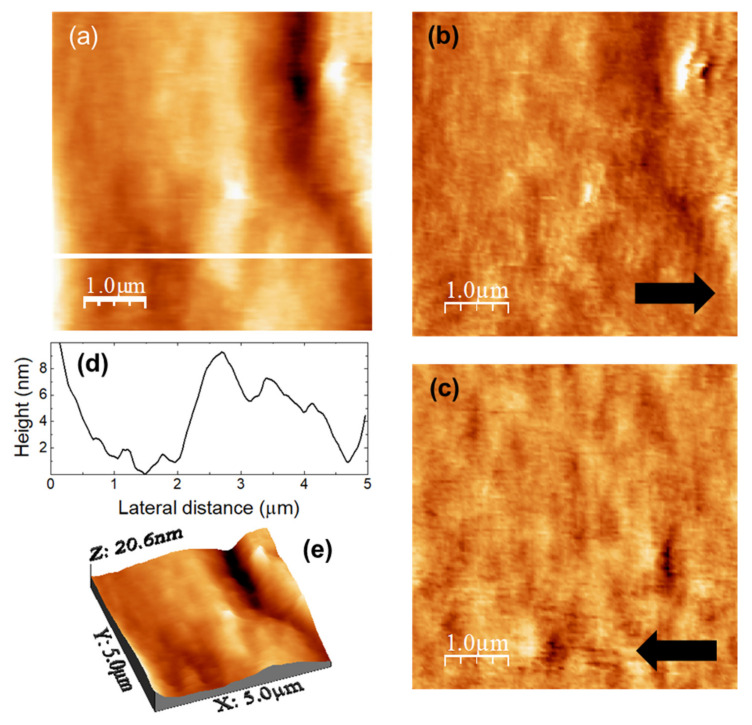
DMA-loaded u-PEO500. (**a**) Contact-mode AFM topography. (**b**,**c**) Lateral force microscopy (LFM) images recorded scanning (**b**) scanning from left to right (simultaneously with (**a**)) and (**c**) from right to left. (**d**) Height-contour profile along the continuous white line in (**a**). (**e**) 3D representation of (**a**).

The images in Figure 4 were recorded over a (5000 × 5000) nm surface area of a DMA-loaded u-PEO film. Figure 4a is the surface topography, and Figure 4b,c lateral force microscopy (LFM) images scanning from right to left (Figure 4b, recorded simultaneously with Figure 4a) and from left to right (Figure 4c, each line recorded as the tip travelled back along the line when recording Figure 4b). Figure 4d shows a height-contour profile along the continuous white line in Figure 4a, and Figure 4e is a 3D representation of Figure 4a. The surface morphology appears slightly more compact than this of the unloaded u-PEO500 film (Figure 3a,e). In Figure 4a,e we may observe a groove, and Figure 4d shows that the areas without groove exhibit a corrugation a bit larger than those of the areas without pores on the unloaded u-PEO surface (Figure 3c). No traces of the presence of DMA can be distinguished on the film surface. The absence of frictional contrast in the LFM images Figure 4b,c confirms the chemical homogeneity of the film surface.

**Figure 5:**
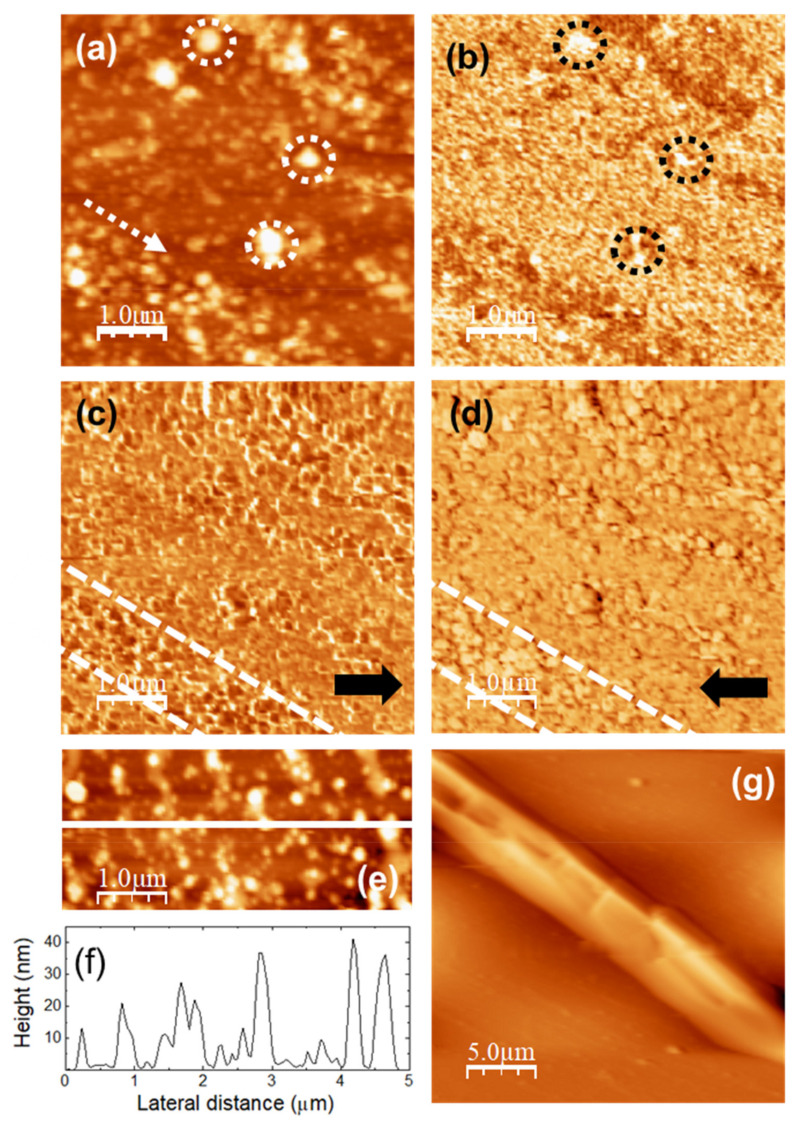
DMA-loaded u-PPO400. (**a**) Contact-mode AFM topography; color-scale range: 64 nm. (**b**) Ultrasonic force microscopy (UFM) image, recorded simultaneously with (**a**). (**c**,**d**) Lateral Force Microscopy (LFM) images recorded scanning (**c**) from left to right, and (**d**) from right to left. (**e**) Contact-mode AFM topography; color-scale range: 55 nm. (**f**) Height-contour profile along the continuous white line in (**e**). (**g**) Contact-mode AFM topography; color-scale range: 2 µm.

In contrast, on DMA-loaded u-PPO films’ surfaces the coexistence of different species or domains is apparent. The images in Figure 5a,b were simultaneously recorded over a (5000 × 5000) nm surface area. Figure 5a is the surface topography, and Figure 5b is the ultrasonic force microscopy (UFM) image, which allows us to distinguish nanoscale regions with different stiffness and/or adhesion [7,32]. The surface morphology in Figure 5a is markedly different from that of the unloaded u-PPO surface (Figure 3b); after loading with DMA, clusters of different sizes can be distinguished on the film surface. The largest clusters in this image, marked with dashed circles in Figure 5a,b are ~300 nm in diameter, and ~80 nm in height. In the UFM image (Figure 5b), such clusters give rise to a brighter contrast, indicative of a higher stiffness. Interestingly, in UFM, sample regions with distinctly darker contrast are clearly noticeable. Even though, in principle, darker contrast in UFM should indicate softer areas, in polymer nanocomposites, depending on the filler/matrix interface properties, the locations of the filler appear with a darker contrast in UFM due to ultrasound scattering at the interface regions [32]. The morphology in those regions (Figure 5a) is characterized by rounded clusters or dots of ~100 nm in diameter, which appear aligned along a well-defined direction, indicated by a dashed white arrow in Figure 5a. Figure 5c,d are LFM images recorded over the same surface area than Figure 5a,b, immediately after these, after suppression of the ultrasonic excitation. In this case, it is clearly noticeable that in the regions in which the UFM contrast appeared darker, such as those between the two dashed white lines outlined in Figure 5c,d, the surface dots in the LFM images exhibit different frictional contrast, with lower friction then their surroundings (LFM image contrast darker when scanning from right to left (Figure 5c), and brighter when scanning from left to right (Figure 5d)). Figure 5e shows a contact-mode topographic image recorded on a different area which also shows the tendency of the dots to align along a well-defined direction; in this image, the coalescence of some dots into segments along that direction is even clearer than in the area in Figure 5a. Figure 5f displays a height contour profile along the continuous white line in Figure 5e, showing that the dots are here smaller than ~200 nm in diameter, and 40 nm in height. Figure 5g correspond to a (25 × 25) µm topographic image recorded over a different surface area of the DMA-loaded u-PPO film. Here, the surface morphology unequivocally reveals the formation of a needle-shaped crystal within the u-PPO film, as a result of the incorporation of DMA. DMA crystalizes in the orthorhombic crystal, and the needle-like crystal in Figure 5g exhibit planes with angles of 90°, characteristics of the orthorhombic morphology. Hence, according to the information provided by the applied AFM techniques, the incorporation of DMA molecules in the u-PPO precursor solution results, after the completion of the sol-gel process, mostly, in the formation of small, rounded clusters or dots, differentiated from the matrix film, which tend to be aligned along a well-defined direction, and, also, in the occasional formation of bigger apparently crystalline needle-like clusters oriented along the same direction.

DMA has a great tendency to form solvents [33]. Two anhydrous polymorphic forms (Form I and II) are known, and two different monohydrated polymorphs have been reported (DEX I and DEX II) [34,35]. In addition, DMA presents an original behavior during its crystallization in specific conditions, and the formation of whisker-like DMA crystals that very much resemble the needle-like crystal observed in Figure 5g corresponding to a sesquihydrated form, has also been reported in the literature, for example, when saturated ethanolic solution of DMA is injected in water, or when DMA crystallized as dimethylsulfoxide (DMSO) solvate is immersed in water [36,37,38]. The mechanism of formation of these whiskers is not yet fully understood, but it is believed to involve a high local saturation that leads to precipitation with dendritic growth. During the sol-gel process, the solvent evaporates while water is formed during the condensation/hydrolysis reactions of the precursors’ molecules, so it is plausible that the formation of the needle-like crystals in Figure 5g originates similarly to the aforementioned DMA whiskers. The observation of these crystals in u-PPO400 is of high interest [39].

DMA has previously been incorporated into bioresorbable films of poly(lactic acid) (PLA) and copolymers of lactic acid and glycolic acid (poly(DL-lactic-co-glycolic acid), (PDLGA)), and the morphology of the loaded films has been studied in detail [16,17]. The films were prepared by solution casting, with the polymer dissolved in a solvent and mixed with the drug prior to casting, followed by isothermal heat treatment after preparation. The DMA location/dispersion in the film was controlled by considering solubility effects in the starting solution and the solvent evaporation rate, which determined the kinetics of drug and polymer solidification. For high drug concentrations in the initial solution, and fast solvent evaporation rates, films with small drug particles and crystals were obtained, with drug nucleation and segregation, as well as merging of the small DMA particles to form larger crystals occurring within the dense polymer solution. In ureasil-polyether films prepared by the sol-gel procedure, the film time formation, and therefore the solvent evaporation time, is relatively fast, faster for unloaded u-PPO than for u-PEO, of the order of ~200 s for precursor molecular weights of 2000 and 1900 g·mol^−1^, respectively [40], and the DMA molecules could behave similarly, allowing us to explain the origin of the small dots observed in DMA-loaded u-PPO films, and their apparent coalescence into segments, noticeable, for instance, in Figure 5e.

**Figure 6:**
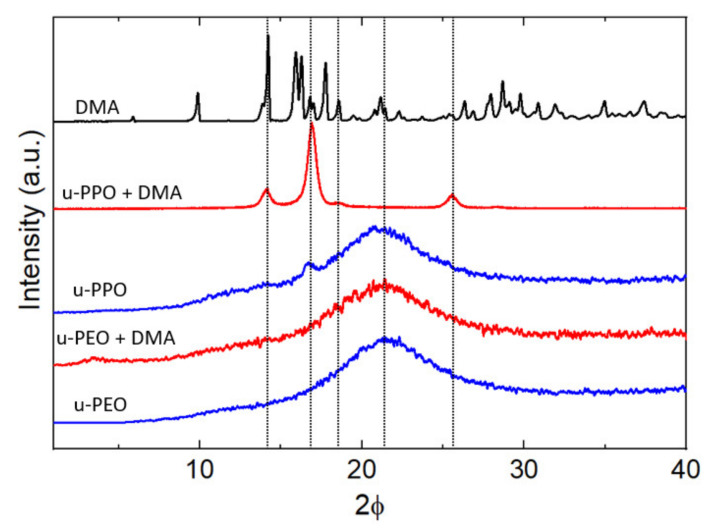
X-ray diffraction patterns for unloaded u-PEO and u-PPO films (blue curves), DMA-loaded u-PEO and u-PPO films (red curves) and DMA powder (black curve).

Figure 6 shows the XRD data recorded on unloaded (blue curves) and DMA-loaded (red curves) u-PEO500 and u-PPO400 films, as well as on the original DMA powders (top black curve). Both unloaded u-PEO and u-PPO films exhibit a broad peak characteristic of amorphous materials with its maximum located at 2θ = 21.6° in u-POE, and 2θ = 21.4° in u-PPO. Interestingly, an additional small peak is apparent in the unloaded u-PPO spectra, located at 2θ = 16.8°, which indicates a small amount of crystallinity. This peak corresponds to an interplanar spacing of 5.2 Å, which coincides with the 200 reflection of the orthorhombic structure of crystalline PPO [41,42]. However, for u-PPO400, the PPO moieties in the precursor molecules are quite short (see Figure 1, n < 7 for the u-PPO precursor), and not necessarily isotactic. This result is quite surprising, since to our best knowledge, even though in some cases coexistence of crystalline and amorphous phases has been observed, for instance, in u-PEO samples obtained from high molecular weight precursors (of molecular weight around 1000 g/mol or higher) [10], no trace of a crystalline phase has been previously observed in ureasil-polyether films obtained from low molecular weight precursors.

The diffractogram of the DMA-loaded u-PEO film does not experience any significant modification with respect to that of the unloaded u-PEO. In contrast, in the DMA-loaded u-PPO sample, the data clearly reveal the presence of a crystalline phase. Table 1 lists the reflections observed in the DMA-loaded u-PEO diffractogram, the corresponding interplanar spacing, and the crystallite size, derived from Debye-Scherrer equation.

It is noticeable from Figure 6 that the peak at 16.8° on the unloaded u-PPO matrix is increased in the DMA-loaded film, and slightly shifted to 16.9°. It happens that this peak is coincident with a reflection from the monohydrated DMA form (DEX I) [34,36]. The peak at 14.0° corresponds to most intense peak in the original DMA powder diffractogram (top black curve in Figure 6) and can be found as a strong peak in the diffractogram of the DMA anhydrous variety (FORM II) [36], but not in this one of the monohydrated phase. Regarding the peak at 25.5°, it is coincident with the 210 reflection of the orthorhombic structure of crystalline PPO [41,42], and it is not present as a strong peak in either the anhydrous or the monohydrated DMA phases. Hence, in view of the experimental data, we cannot reject the possibility that a crystalline PPO phase forms within the u-PPO matrix, enhanced by the presence of DMA, which also appears to be present in crystalline state.

At first sight, it is surprising that the peaks of the sesquihydrate phase of DMA, which should correspond to the needle-like crystals in Figure 5g, considering its resemblance to the DMA whiskers, do not appear in the XRD diagram. We attribute this to the fact that this crystal phase is forming in small quantity, and just at the sample surface, while the information recorded in XRD comes from a surface region of several microns.

For the application of the Debye-Scherrer equation that allows us to estimate the crystallite sizes of the present phases we have considered the dimensionless shape factor K as 0.94, i.e., presuming that the domains have rounded shape. According to the morphology in Figure 5a,e this should indeed be the case. The obtained data of ~27 nm for the crystallite size is also consistent with the AFM images in Figure 5. Even though the largest clusters in Figure 5a reach up to ~300 nm, and in Figure 5e ~100 nm, there are many smaller dots of ~30 nm in size. Furthermore, the ones that we may appreciate in the image are located on the film surface, and it is to be expected that the ones located below are probably somewhat smaller, being constrained by the surrounding matrix.

**Figure 7:**
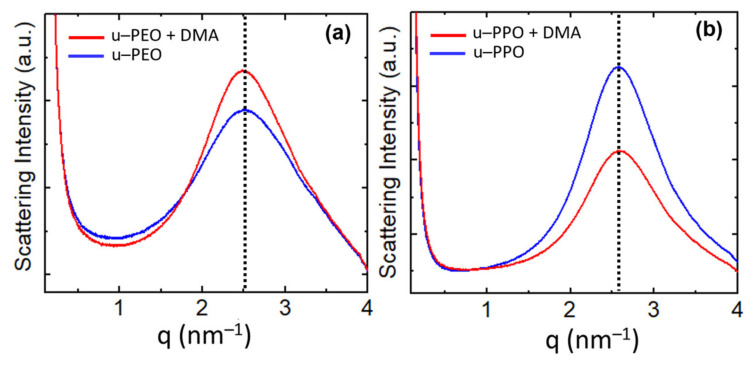
Experimental SAXS intensity *I(q)* of (**a**) unloaded u-PEO 500 (blue curve) and DMA-loaded u-PEO500 (red curve) and (**b**) unloaded u-PPO (blue curve) and DMA-loaded u-PPO400 (red curve).

Figure 7 shows the SAXS curves recorded on (a) u-PEO500 and (b) u-POP400. The blue curves correspond to unloaded samples and the red to samples loaded with DMA. The SAXS intensity, I(q), is proportional to the Fourier transform of the correlation function of the electronic density of the material. The peaks give evidence of a strong spatial correlation between the ureasil-polyether cross-linked siloxane nodes. For both unloaded u-PEO and u-PPO the maximum peak position is located at $q=2.5\;{\mathrm{nm}}^{-1}$, and the averaged most probable distances between two adjacent siloxane nodes within the polymer matrix can be estimated by $d=\frac{2\pi }{q_{max}}=2.5\;\mathrm{nm}$, where *q_max_* is the modulus of the scattering vector at the peak’s maximum. As seen in Figure 7, the introduction of DEXA does not significantly alter this distance, the maxima of the red curves in Figure 7a,b remain approximately in the same position as those of the blue ones. An average size of the correlation volume associated with the spatial distribution of siloxane nodes, *Lc*, can be obtained by applying the Scherrer equation in the case of low-angle X-ray scattering ($L_{c}=4\pi /\Delta q$) where Δ*q* is the full width at half-maximum of the correlation peak of the SAXS function [43,44]. The values of *Lc* for the SAXS peaks in Figure 7 are ~9.0 nm for the unloaded and DMA-loaded u-PEO films, and ~12.5 nm for the unloaded and DMA-loaded u-PPO films.

Interestingly, as DMA is introduced in u-PEO, the intensity of the correlation peak increases, indicating the increase of the electronic-density contrast between the ureasil nodes and the polymeric matrix, as observed in other cases [1,43]. On the other hand, in u-PPO films, the intensity decreases.

**Figure 8:**
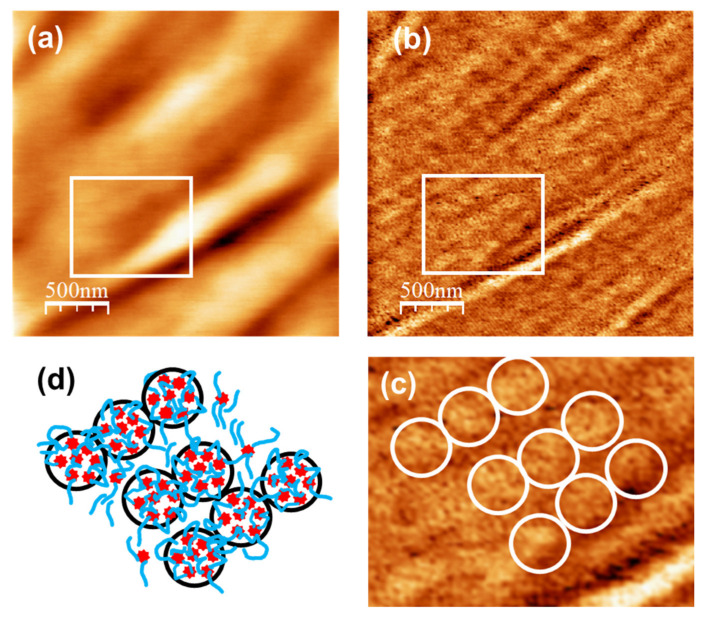
Unloaded u-PPO400. (**a**) Contact-mode AFM topography; color-scale range: 64 nm (**b**) Ultrasonic force microscopy (UFM) image, recorded simultaneously with (**a**); (**c**) Cropping of the part of (**b**) indicated by the white rectangle (**d**) Schematic drawing to illustrate the nanostructural arrangement within the polymer matrix.

Figure 8a,b corresponds to AFM and UFM images recorded over a (2500 × 2500) nm surface area on the unloaded u-PPO film. Figure 8a is the surface topography recorded in contact-mode and Figure 8b is the UFM image recorded simultaneously with Figure 8a, over the same surface area. As Figure 3b,f, Figure 8a shows that the u-PPO400 matrix is formed by polymer ropes, oriented along a well-defined direction. The UFM image (Figure 8b) makes it possible to distinguish that the ropes are formed by “strands” ~180 nm in diameter, which in turn are structured in rounded clusters. Figure 8c is a crop of the area delimited by the white rectangle in Figure 8b, in which some rounded clusters have been enclosed by white circles to facilitate their identification. The clusters yield a brighter contrast in UFM, which indicates that they correspond to stiffer regions. The contrast in Figure 8b can be understood if the siloxane nodes within the u-PPO400 matrix assemble into “hybrid clusters”, ~180 nm in diameter, corresponding to the rounded clusters seen in the image. In fact, according to the information provided by SAXS (Figure 7), each of these clusters should be formed by ~14 (i.e. (cluster diameter)/*Lc*) disordered hybrid “supercrystals” defined by aggregates of siloxane nodes with average separation distances of ~2.5 nm between them.

Figure 8d depicts a tentative sketch of the u-PPO400 matrix structure, in which the red stars represent the hybrid supercrystals of siloxane nodes aggregates that give rise to the SAXS correlation peak in Figure 7b.

The model in Figure 8d provides insight into the results of Figure 5. The new crystalline phases formed within the DMA-loaded u-PPO400 matrix may occupy the sites in-between the strands defined by the aligned clusters in Figure 8d. Furthermore, the “stiffer clusters” observed on the surface of the DMA-loaded u-PPO400, (those enclosed with dashed circles in Figure 5a,b), surely correspond to some of the clusters in Figure 8b, displaced from their sites as the new phases formed upon drug loading.

**Figure 9:**
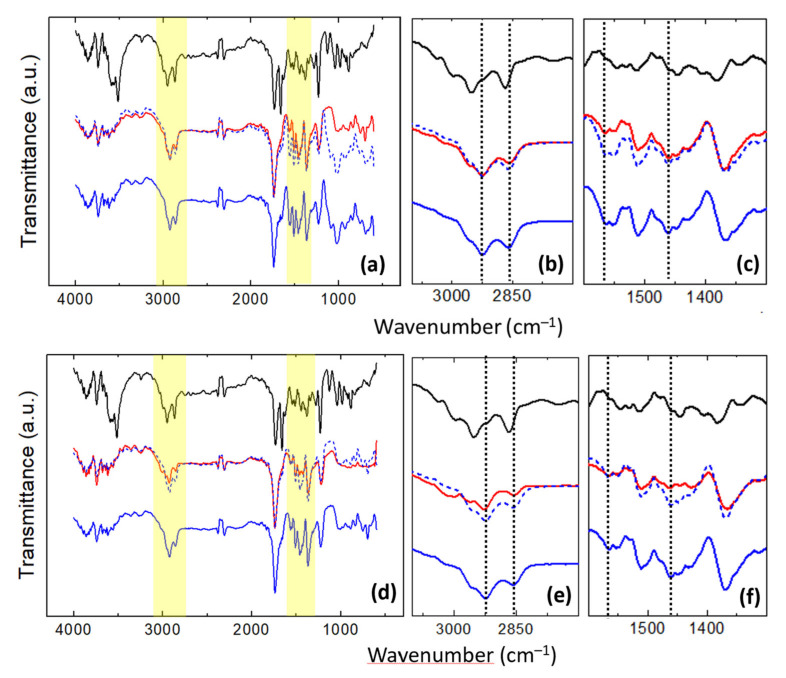
FT-IR spectra of (**a**–**c**) pure u-PEO 500 films (blue curves), DMA loaded u-PEO films (red curves) and DMA powder (black curves); and (**d**–**f**) pure u-PPO400 films (blue curves), DMA loaded u-PPO films (red curves) and DMA powder (black curves). In each plot, the dashed blue curve is identical to the continuous blue line and has been shifted vertically to facilitate the comparison of the unloaded and DMA-loaded films’ spectra.

Figure 9 displays the FT-IR spectra recorded on unloaded (blue curves) and DMA-loaded (red curves) (Figure 9a–c) u-PEO and (Figure 9d–f) u-PPO films. The black curves in Figure 9 correspond to the FT-IR spectrum recorded on the original DMA powders.

Table 2 lists the main vibrational peaks observed in the FT-IR spectra and their assignments [4,34,45]. Due to the similarity of the functional groups, most of the characteristic vibrational bands of the ureasil-polyether matrix and the DMA molecules occur in the same regions.

For the discussion of the information provided by the FT-IR data we will mainly focus on the yellow regions in Figure 9a,d enlarged in Figure 9b,c,e,f.

Figure 9b,e shows the C-H stretching region. Here, in both unloaded u-PEO and u-PPO curves (blue curves in Figure 9b,e), the peaks at 2925 cm^−1^ and 2859 cm^−1^, assigned to CH_2_ antisymmetric and symmetric stretching, respectively [4], have been indicated with a vertical dashed line. In the original DMA powder spectrum (black curves in Figure 9b,e), we encounter several bands in this region corresponding to olefinic and aliphatic CH stretching [45]. In DMA-loaded u-PEO films, the incorporation of DMA to the matrix does not bring much change in the FT-IR bands (see red curve in Figure 9b). Nevertheless, in DMA-loaded u-PPO films, the incorporation of DMA is accompanied by a reduction in the CH_2_ stretching vibrations observed in the unloaded samples, and by the emergence of a small peak at 3020 cm^−1^ which can be related to olefinic CH stretching from the DMA molecules (see red curve in Figure 9e). The fact that the CH_2_ stretching vibrations diminish confirm that the u-PPO structure is being altered in the presence of DMA, which is in agreement with the results obtained from the AFM and XRD studies.

Figure 9c,f includes the amide and CH_2_/CH_3_ absorption bands. The peak at 1564 cm^−1^ indicated by a vertical dashed line, discernible in both spectra of the unloaded u-PEO and u-PPO films (see blue curves in Figure 9c,f), has been previously assigned to the amide II band [4]. The amide II mode is a mixed contribution of the N-H in-plane bending, the C-N stretching and the C-C stretching vibrations, and it is very sensitive to both chain conformation and intermolecular hydrogen bonding. As it is well known, the urea compounds have a very strong self-association capacity, and in small molecular weight ureasil-polyether, the formation of many urea-urea linkages is expected. The N-H groups of the urea moieties are prone to form strong hydrogen bonds with either the carbonyls of the urea moieties of another molecules, and/or with the ether oxygens of the polyether moieties, capable to act as hydrogen bond acceptors. Interestingly, in DMA-loaded u-PEO film this peak strongly diminishes (Figure 9c), indicating that the presence of DMA in u-PEO alters the interactions of the N-H that exist in the unloaded matrix.

Consistent with our previous observation, the peak at 1100 cm^−1^ in the u-PEO film (Figure 9a, blue curve) which falls into the COC stretching region [46], strongly diminishes when DMA is incorporated (Figure 9a, red curve). In contrast, the amide II band does not experience any changes in u-PPO films when they are loaded with DMA (Figure 9f). In u-PPO, due to the presence of the additional CH_3_ group, it is conceivable that the DMA molecules cannot easily access the ether oxygens of the polypropylene moieties. DMA may interact with the u-PPO matrix at sites near the ureasil nodes but if the DMA molecules reach the sites amidst the strands of clusters in Figure 8, where there are no ureasil nodes, they are bound to interact with each other and form the new phases observed by both XRD and AFM. The fact that u-PPO is structured in strands formed by aligned clusters which consist in aggregates of siloxane-nodes, as illustrated in Figure 8, explains that the new DMA phases form along a well-oriented direction, growing between the cluster strands.

The peak at 1460 cm^−1^, also marked with a vertical dashed line and noticeable in both spectra of the unloaded u-PEO and u-PPO films (blue curves in Figure 9c,f) is assigned to a C-H mode. Consistent with the results discussed in relation to Figure 9b,e, this band experience no changes when u-PEO is loaded with DMA, but it is severely affected (the intensity of the band is reduced, i.e., the vibrational modes are hindered) when DMA is incorporated into the u-PPO films.

**Figure 10:**
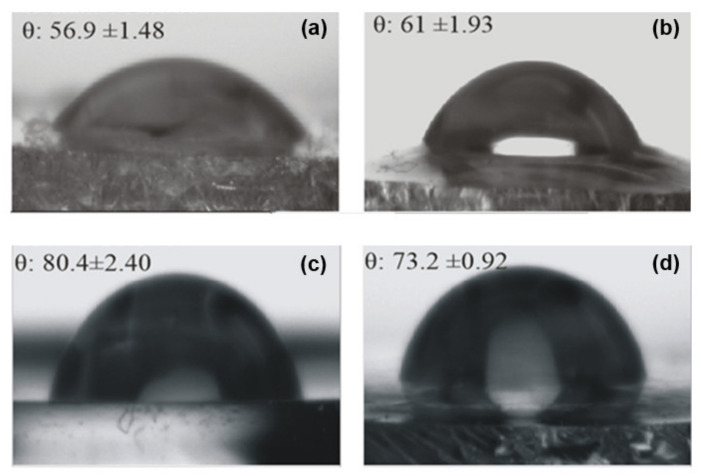
Contact-angle measurements on (**a**) unloaded u-PEO500; (**b**) DMA-loaded u-PEO500; (**c**) unloaded u-PPO400; (**d**) DMA-loaded u-PPO400. The results are expressed as the mean ± S.D. for n = 3 (replicates).

Figure 10 presents the values obtained from contact-angle measurements (θ) on the ureasil-polyether films’ surfaces, together with the recorded optical images. The comparison of the contact angle of the unloaded u-PEO500 and u-PPO400 films indicates that the value is significantly larger for u-PPO, which is consistent with its higher hydrophobic character. Due to its higher hydrophobic nature, u-PPO has lower affinity with water and therefore exhibits a higher contact-angle value. The incorporation of DMA induces changes in the contact angle values in both ureasil-polyether films.

In the DMA-loaded u-PEO500 films, the contact angle increases as a result of the addition of DMA. According to the AFM results (Figure 4), no traces of DMA are found on the loaded u-PEO films surfaces. Nevertheless, as indicated by FT-IR (Figure 9), DMA does cause modifications in the u-PEO matrix by hindering the interactions between amide and ether oxygen from different polymer chains. If DMA and the poly(oxyethylene) moieties form complexes involving the ether oxygen, the presence of DMA may facilitate the elimination of water produced during the sol-gel reactions outside the hybrid film and reduce the number of reactive sites at surface locations in such a way that the resulting film surface exhibits reduced hydrophilicity.

In the case of u-PPO400 films, the addition of DMA to the polymer matrix diminishes the contact-angle value (i.e., increases the surface hydrophilicity). DMA is considered a hydrophobic molecule, with a low solubility in water. Nevertheless, the result is understandable considering that the structure of the u-PPO film is severely disrupted in the presence of DMA, with the formation of new crystalline phases. The increased hydrophilicity in DMA-loaded u-PPO400 film is bound to result in an increased bioadhesion of the film, with an increased probability of H-bonds formation on the film surface [13].

**Figure 11:**
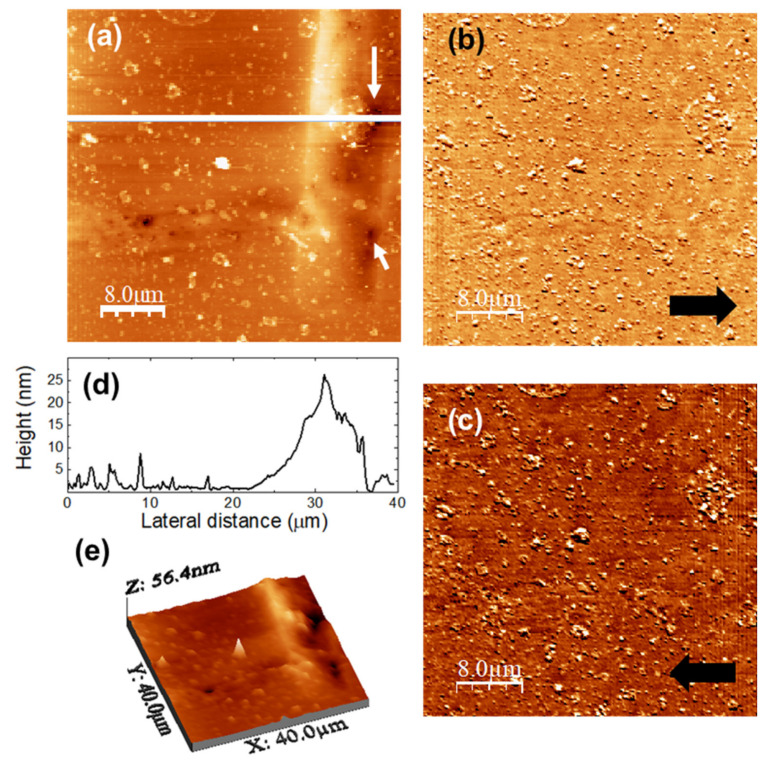
u-PPO500 film after DMA release in medium. (**a**) Contact-mode AFM topography; color-scale range: 35 nm. (**b**,**c**) Lateral force microscopy (LFM) images recorded (**b**) scanning from left to right (simultaneously with (**a**)) and (**c**) from right to left. (**d**) Height-contours profile along the continuous white line in (**a**). (**e**) 3D representation of (**a**).

To evaluate surface modifications as a result of DMA release from u-PPO films, we immersed the samples in a solution containing 500 mL of medium (phosphate buffer 7.2 pH with 0.5% of procetyl AWS^®^ (CRODA, Rawcliffe Bridge, UK)) in a manner similar to the drug release experiments reported in Figure 2, for 24 h, to ensure that little or no drug remained to be released. The images in Figure 11 were recorded over a (40,000 × 40,000) nm surface area of the u-PPO film after release of DMA into the medium. Figure 11a is the surface topography, and Figure 11b,c lateral force microscopy (LFM) images scanning from right to left (Figure 11b, recorded simultaneously with Figure 11a) and from left to right (Figure 11c, each line recorded as the tip travelled back along the line when recording Figure 11b). Figure 11d shows a height-contour profile along the continuous white line in Figure 11a. Figure 11e is a 3D representation of Figure 11a. The absence of frictional contrast in the LFM images Figure 11b,c confirms the chemical homogeneity of the film surface, i.e., the absence of DMA-related clusters on the film surface.

The surface morphology supports the information provided by the analysis of the release kinetics (Figure 2), according to which DMA release from u-PPO matrices mostly occurs by Fickian diffusion. No sign of erosion or swelling of the matrix substrate is apparent. The morphology is characterized by the presence of small clusters, typically lower than ~10 nm in high. The ~20 nm rectangular protrusion on the right-hand side of the image clearly indicates the previous location of a DMA crystal. Precisely at the protrusion sides, some small pores are apparent, such as those indicated by the white arrows in Figure 11a, also clearly noticeable in Figure 11e, which presumably form as the drug is released. Apparently, the molecular chains of the polymer matrix accompany the drug and re-form, as the drug diffuses outwards, leaving the matrix substrate.

## 4. Conclusions

The study we have carried out has allowed us to deepen our understanding of the nanostructural arrangements within ureasil-polyether matrices and how they interact with DMA drug molecules.

Key points disclosed by this work are the following:

- In u-PEO500, DMA occupies sites near the ether oxygen and remains absent from the film surface. The release kinetics of DMA from the u-PEO500 matrix fits well with Peppas’ model; the formation of pores on u-PEO500 may enable the penetration of water inside the matrix and its swelling to facilitate the release process.
- In u-PPO400, new crystalline phases are formed upon loading with DMA, which show up as rounded clusters of ~30–100 nm in diameter, aligned along a well-defined direction. The study of the u-PPO400 matrix shows that it is structured in polymer ropes, composed of strands, consisting of aligned clusters of ~180 nm in diameter, formed by aggregates of ureasil-nodes supercrystals. Hence, the new phases may grow between the strands, where ureasil nodes are absent. The formation of larger needle-shaped crystals has also been observed. The release kinetic of DMA from the u-PPO400 matrix fits well with Weibul model; when DMA release comes to an end, the matrix surface shows no trace of DMA and no evidence of erosion.

The results indicated above extend our current knowledge on ureasil-polyether, and DMA interactions within these matrices. In our opinion, the most relevant contribution of this work is the observation by means of UFM of aligned clusters of ureasil-node aggregates defining hybrid-polymer strands. UFM is a relatively new technique, and, in many areas, its full potential has not yet been explored. To our knowledge, no such structuring of uresil nodes has been observed before, and it should play a decisive role in many of the diverse applications of these hybrid polymer matrices. We believe it is likely that u-PEO500 is also structured into strands formed by aligned clusters of ureasil-node aggregates, but the presence of pores favors water penetration into these matrices, and swelling hinders their observation by AFM/UFM procedures under ambient conditions.

## Figures and Tables

**Table 1:** Peaks in the DMA loaded u-PPO diffractogram.

2θ/Degrees	d-Spacing (Å)	Crystallite Size (nm)
14.0094	6.3163	26.11
16.9142	5.2375	27.19
25.5421	3.4845	27.58

**Table 2:** Peak assignment in the FT-IR spectra in Figure 9.

FT-IR Wavenumber (cm^−1^)	Peak Assignment
3516	O-H stretching
3050–2850	C-H stretching
1740, 1660	C=O stretching
1565	Amide II
1475–1427	CH_2_ scissoring/CH_3_ deformation
1370–1351	CH_2_ wagging
1100	C-O stretching

## Data Availability

The data presented in this study are available on request from the corresponding author.
